# Unmet needs of activities of daily living among a community-based sample of disabled elderly people in Eastern China: a cross-sectional study

**DOI:** 10.1186/s12877-018-0856-6

**Published:** 2018-07-11

**Authors:** Shen Chen, Jing Zheng, Chen Chen, Ying Xing, Yan Cui, Yaping Ding, Xiuyun Li

**Affiliations:** 1 School of Nursing, Nanjing Medical University, 101 Longmian Avenue, Jiangning District, Nanjing, 211166 People’s Republic of China; 2 Nanjing Health Service Center of Mofan West Road, No.3 Dinghuaimen, Nanjing, 210013 China

**Keywords:** Unmet needs, Activities of daily living, Aged, Disability, China

## Abstract

**Background:**

China has the largest population of partially or completely disabled elderly people in the world. Although the disabled elderly people try to remain independent in their lives, many still need assistance from others. Failure to obtain sufficient assistance creates a situation of unmet need. Unmet needs of activities of daily living (ADL) for disabled elderly people pose significant risks for hospitalization and mortality and cause an increased economic burden on families and society. This study aimed to identify the prevalence and risk factors of unmet needs among the disabled elderly in China to guide government toward corrective action.

**Methods:**

A total of 303 older adults from 15 communities in Nanjing, China were recruited. The Barthel Index (BI) and Functional Activities Questionnaire (FAQ) were used to screen disabled elderly people from the communities. These disabled elderly participants were then investigated in terms of their unmet ADL needs, using an unmet needs assessment form, which had been adapted from the BI and FAQ. Additionally, the Zarit Burden Interview and Family Caregiver Task Inventory were used to survey the main caregivers. Finally, univariate analysis was first used to filter out candidate impact factors, and then, binary logistic regression analysis was used to adjust for cofounders and determine reliable risk factors.

**Results:**

A total of 93.1% of the disabled elderly people in our study reported at least one unmet need. The prevalence of unmet needs for different ADL tasks ranged from 4.6 to 77.2%. The unmet needs with the highest percentages were using vehicles (77.2%), using stairs (73.1%), working on a hobby (72.1%), social interaction (62.6%) and ambulating (60.1%). The factors influencing unmet needs were related to the degree of disability in instrumental activities of daily living (IADL) (OR = 1.079, *p* ≤ 0.01), the relationship with caregivers (OR = 1.429, *p* ≤ 0.05) and the monthly income of caregivers (OR = 0.679, p ≤ 0.05).

**Conclusion:**

Disabled elderly people living in communities had a high percentage of unmet needs for activities of daily life that required going outside the bedroom and involved spiritual aspects. Unmet needs increased with worsening disability status in IADL, more distanced relationships with caregivers and lower incomes of caregivers. Both government and caregivers should take more action to prevent or reduce unmet needs among the elderly.

**Electronic supplementary material:**

The online version of this article (10.1186/s12877-018-0856-6) contains supplementary material, which is available to authorized users.

## Background

The unprecedented and pervasive process of population aging has become a global challenge that no longer exists only in developed countries [1]. Because of the “One Child Policy” adopted in 1979 as well as economic development and advances in medical technology, China became an “aging society” in approximately the year 2000 [2]. The latest data published by the National Bureau of Statistics of China indicated that more than 143.86 million people were over 65 years old in 2015, approximately 10.47% of the total population of China [3].

Along with the acceleration of aging, the number of disabled elderly people is also rapidly increasing. The International Classification of Functioning, Disability and Health (ICF) defines disability as an umbrella term for impairments, activity limitations and participation restrictions [4]. Completely disabled persons and partially disabled persons with functional deficits due to age, disease or accidents are all considered disabled in this study. Evaluating basic activities of daily living (BADL) and instrumental activities of daily living (IADL) is considered the most common way to screen for disabilities in the elderly [5]. Statistics investigated by the Research Group of China Research Center on Aging show that by 2010, China had more than 22.15 million partially disabled and 10.84 million completely disabled elderly people (over 60 years old), accounting for 12.75 and 6.25%, respectively, of the total elderly population [6]. Many studies have explained the relationships between aging and disability. One theory is that due to a decline in mortality rates, the onset of disability will be postponed until late in life and thus will be “compressed” for elderly people who live with disability [7]. Studies also suggested that chronic diseases, such as hypertension, diabetes and cerebrovascular disease, common in the elderly, may result in disability [8, 9]. Multimorbidity among the elderly, which is a world-wide public health issue, is significantly associated with disability [10, 11].

Disability can cause many adverse consequences, such as a decline in quality of life and physical or mental health and an increased risk of harm from accidents [12, 13]. However, as a developing country with a large disabled aging population, China has an even larger challenge in caring for aging adults: unlike Japan and Germany, China does not have a social nursing insurance system. Thus, the disabled elderly people cannot receive additional insurance money to support their everyday lives. In China, only when the elderly suffer from severe organic diseases will costs be partly payed by medical insurance. Similar to many other developed countries, China has already established a basic medical insurance system, which covers approximately 95% of the total population [14]. However, not everyone can receive high-quality medical services without worrying about money. China has 3 types of social medical insurance systems [15]. Less than 20% of the elderly have Basic Medical Insurance for Urban Employees, where they do not have to pay any insurance fees after retirement and can get the highest reimbursement ratio of medical costs. The rest of the elderly either have Basic Medical Insurance for Urban Residents (Freelancers and the unemployed) or New Rural Cooperative Medical Insurance, which requires continued payment of insurance fees, even after retirement, and has more limitations on receiving medical services. In contrast to western countries, the wealthy in China seldom donate money to support the disabled elderly, and subsidies from government are very low. Although the elderly living in some large cities have experienced substantial improvement in disability treatment due to additional resources for caregiving over the past decade, most elderly people still live in a situation without long-term care and specific insurance [16]. Nursing homes in China are still at the preliminary stage with only approximately 6.72 million beds available in the entire country [17]. Hence, most disabled elderly people are cared for by their family members, especially women [18]. However, traditional caregivers experience a much heavier burden than previously, as there are more and more nuclear families (a couple with an unmarried child) and women with careers in China [19].

Although the disabled elderly try to remain independent in BADL/IADL, some may still need assistance from others. Failure to obtain sufficient assistance creates a situation of unmet need [20]. Kim defines unmet need as “the gap between the amount of long-term care need, as assessed by an individual, and the actual resources the individual has at his/her disposal to meet that need” [21]. Unmet needs occur when assistance is not provided or is inadequate [22]. Unmet need is usually measured by self-report, but sometimes, caregivers and care-recipients share their different attitudes toward perceived unmet needs for services [23]. There are studies available that indicate that unmet needs among disabled elderly people lead to adverse consequences, including higher risks of hospitalization, hospital readmission, emergency department admission, psychological distress and death [24–27]. To reduce the lack of individualized care, some countries have established long-term care systems. Japan and Germany are famous for their universal coverage of long-term care systems with services provided by non-profit organizations [28]. These systems are financed through government, employers and employee contributions. The United States also has an excellent system with services provided by for-profit organizations, including adult day service centers, home health agencies, nursing homes and residential care communities [29]. However, for these, the elderly need commercial insurance.

Most previous studies related to unmet need were conducted in developed countries, but the prevalence of unmet needs varied widely in different countries, regions and races. Compared with other countries, China has large numbers of disabled elderly people and has different cultures, policies and lifestyles, so it is urgent to investigate the problems of unmet need in China, where such types of studies are rare. China may not have an optimistic result regarding unmet needs because the healthcare system in China is still not very efficient. Therefore, the present study aims to identify the prevalence and risk factors of unmet need among the Chinese disabled elderly. The findings may be helpful for government and caregivers to take more useful action to prevent or reduce unmet needs. Thus, disabled elderly people can have the chance to improve or maintain an optimal level of physical functioning and quality of life.

## Methods

The study protocol received approval by the Ethics Committee of Nanjing Medical University with approval no. 2017–579.

### Data source and analytic sample

Data for this study were collected from 15 communities in 7 districts of Nanjing city, Jiangsu Province, which is located in the coastal area of Eastern China. These communities are scattered in urban, suburban and rural areas. Individuals included in this study were selected from the electronic health systems in different communities with an oral agreement to participate in the study. Criteria for inclusion in the study were: age over 60 years old; meeting the disability criteria of the Barthel Index (score ≤ 60) or Functional Activities Questionnaire (score ≥ 5); ability to answer questions or have caregivers who are familiar with the situation answer questions. Elderly people with intellectual disabilities were excluded from this study. The sample size for this study was calculated as 291 using the formula: N = μ_α_^2^ × π(1-π)/δ^2^. In this formula, μ_α_ = 1.96, δ = 0.1π, and π = 56.88% (according to previous studies [30]). The final sample yielded 303 community-dwelling disabled elderly people who met the criteria.

### Instruments

**Fig. 1:**
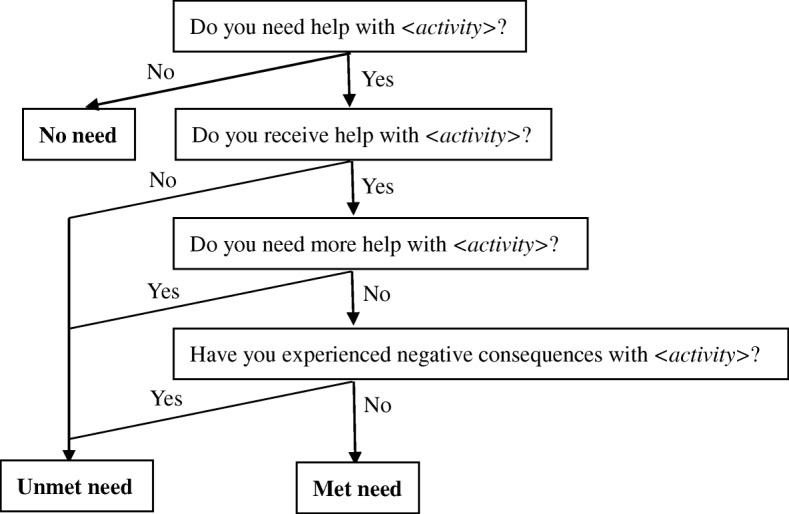
Determination of assistance need status for ADL

Several questionnaires were used to measure different variables in this cross-sectional study, including the following:

1. Demographic questionnaire (Additional file 1): We designed this questionnaire ourselves; it was divided into two parts according to care recipient or caregiver. The care recipient section contains age, gender, marital status, education, income, medical expenses, and cause and length of disability of the disabled elderly person. The second section contains age, gender, education, relationship with the care recipient and income of main caregivers.
2. Barthel Index (BI) (Additional file 2): The BI was modified by Mahoney and Barthel in 1965 [31] and is used for measuring the functional status of BADL, which consists of feeding, dressing, bathing, grooming, toileting, bowel control, bladder control, chair/bed transfer, ambulating, and using stairs. Scores range from 0 to 100 to identify the severity of disability in BADL; the higher the score, the lower the level of independence. Scores are classified into 5 groups: 0–19 is complete dependency, 20–40 is severe dependency, 41–60 is moderate dependency, 61–99 is mild dependency, and 100 is no dependency. Participants can be defined as disabled in BADL when the total score is ≤60.
3. Functional Activities Questionnaire (FAQ) (Additional file 3): The FAQ was modified by Pfeffer in 1982 [32], and is used for measuring the functional status of IADL. It has 10 questions, which can be abbreviated as financial management, assembling affairs, shopping, working on a hobby, doing housework, cooking, learning about current events, communicating, memorizing, and using vehicles. Each item’s score ranges from 0 to 3, from independence to dependence. Participants can be defined as disabled in IADL when the total score is ≥5.
4. Zarit Burden Interview (ZBI) (Additional file 4): The ZBI was modified by Zarit in 1980 [33] and is used to measure caregiver burden. The revised version contains 22 items. Each item in the interview is a statement that the caregiver is asked to respond to using a 5-point scale. Response options range from 0 (Never) to 4 (Nearly Always). The higher the total score, the heavier the burden.
5. Family Caregiver Task Inventory (FCTI) (Additional file 5): The FCTI was originally developed by Clark in 1983 [34], was then translated into the Chinese version by Lee from Hong Kong in 2004 [35]; it is used for measuring difficulties in caregiving. The instrument includes 25 items answered on a scale of 0–2, ranging from no difficulty to difficulty. The higher the total score, the more difficulty in caring for care-recipients.
6. Unmet Needs Assessment (Additional file 6): This tool is a 17-item questionnaire based on perceived need and was adapted from an algorithm created by Allen and Mor in 1997 (see Fig. 1) [36, 37]. Using this tool, unmet need can be filtered according to 3 principles: Are you receiving someone’s help? Is the present assistance enough? Were there any negative consequences during the past month? Questions used to assess the negative consequences of unmet needs are shown in Table 1. Investigators can conduct interviews with disabled elderly people following the guidelines of this assessment. If respondents did not get help, did not get enough help or experienced any negative consequences in a specific activity, this activity can be considered an unmet need. All items in this questionnaire were picked up from the BI and FAQ with using the bathroom, bowel control and bladder control combined as toileting and learning about current events and communicating summarized as social interaction. The 17 items include feeding, dressing, bathing, grooming, toileting, chair/bed transfer, ambulating, using stairs, financial management, assembling affairs, shopping, working on a hobby, doing housework, cooking, social interaction, memorizing and using vehicles. Of all 17 items, feeding is a special one. If someone cannot eat on their own, he/she should have help from others; otherwise, he/she could die soon and have no chance to participate in this study. Thus, investigators should only identify whether he/she has enough help or negative consequences.

**Table 1:** Questions used to assess negative consequences of unmet needs for ADL tasks

Items	Questions (during the past month)
Feeding	Were there times you were unable to eat when you were hungry because no one was available to help you have food?
Dressing	Did you experience discomfort because you were unable to get assistance to change your dirty or wet clothes as soon as possible?
Bathing	Did you experience discomfort because you were unable to get assistance to bathe as often as possible? Did you experience a scald caused by bathing with water that was too hot?
Grooming	Did you experience discomfort with your face/teeth/hair because you were unable to get assistance to groom as often as possible?
Toileting	Were there times you had no choice but to hold back urine for a long time because no one was available to help you go to the restroom? Did you wet or soil yourself because you did not have help getting to the restroom?
Chair/bed transfer	Were there times you had no choice but to lie on the bed for a long time because no one was available to help you get out of bed? Have you ever had pressure sores?
Ambulating	Were there times you had no choice but to stay in the bedroom for a long time because no one was available to help you walk outside?
Using stairs	Were there times you had no choice but to stay on the same floor for a long time because no one was available to help you go downstairs?
Financial management	Were there times you lost money or overspent because no one was available to help you do financial management?
Assembling affairs	Did you experience chaos in everyday life because you were unable to get assistance to assemble affairs?
Shopping	Did you experience inconvenience due to a lack of daily supplies because you were unable to get assistance to do shopping?
Working on a hobby	Were there times you felt very bored because no one was available to help you do recreational activities?
Doing housework	Were there times you had no choice but to live in a dirty environment, because no one was available to help you do housework?
Cooking	Did you experience discomfort because you often had to eat food that did not meet your taste?
Social interaction	Were there times you had no choice but to live alone for a long time because no one was available to help you go outside to chat with others?
Memorizing	Were there times you forgot to take medicine or misused medicine because no one was available to remind you to take the medicine?
Using vehicles	Were there times you had no choice but to stay home when there was an urgent issue that needed to be dealt with outside because no one was available to take you to travel some distance?

Chinese versions of the BI, FAQ, ZBI and FCTI have been widely used and are all proven to have good reliability and validity in local articles [38].

### Procedure

Fifteen nursing professionals and graduate students underwent a standard training and a 3-day pre-survey prior to commencement of the study to ensure that every investigator was qualified in his/her work. Then, from December 2016 to July 2017, face-to-face interviews were conducted at participants’ homes by at least 2 investigators to ensure the quality of each investigation. BI and FAQ were used first to confirm that selected individuals were in fact disabled. The status of disability can also be measured by these 2 instruments. Afterward, other instruments were used with participants and their caregivers.

### Data analysis

Data analysis was performed using the SPSS 18.0 software package. Frequency was used to describe the disability status and unmet needs of disabled elderly participants and was also used to describe the characteristics of both care recipients and caregivers, together with means and standard deviations. Chi-squared and Wilcoxon tests were employed for qualitative data and non-normally distributed quantitative data, respectively, to examine the association between unmet needs and characteristics. Finally, a binary logistic regression analysis was used to identify significant risk factors for unmet needs, and confounders were also adjusted in this step.

## Results

### Characteristics

As Table 2 shows, the mean age of the respondents was 82.3 (SD = 9.5) years, and ages ranged from 60 to 109 years. Of the 303 disabled elderly people, slightly more females (54.4%) participated than males (45.5%), and the ratio of married to unmarried, which also includes divorced or widowed, was approximately 1:1. Nearly 60% of the elderly had only primary school-level education or below, 39.9% received less than 1000 yuan per month (approximately 158.7 USD, April 10, 2018) from their insurance or other source of finances, and their medical expenses were also less than 1000 yuan per month. Close to 72% of the individuals suffered from disability because of disease, and 68.7% had been considered disabled for more than 24 months.

**Table 2:** Demographic characteristics of disabled elderly participants and their main caregivers (*N* = 303)

Variables	n	%
Disabled elderly people		
Age(year)		
Mean(SD)	82.3(9.5)	
Gender		
Male	138	45.5
Female	165	54.5
Marital status		
Unmarried	150	49.5
Married	153	50.5
Living area		
Urban	150	49.5
Suburban	121	39.9
Rural	32	10.6
Education		
Illiterate	115	38.0
Primary school	69	22.8
Junior high school	38	12.5
Senior high school	40	13.2
College and above	41	13.5
Income (month)		
≤ 1000 yuan	121	39.9
1001–3000 yuan	51	16.8
3001–5000 yuan	69	22.8
>5000 yuan	62	20.5
Medical expense (month)		
≤ 1000 yuan	177	58.4
1001–3000 yuan	39	12.9
3001–5000 yuan	34	11.2
>5000 yuan	53	17.5
Cause of disability		
Diseases	218	71.9
Accidents	39	12.9
Caducity	46	15.2
Length of disability		
≤12 months	34	11.2
13–24 months	61	20.1
>24 months	208	68.7
Main caregivers		
Age (year)		
Mean(SD)	59.0(12.7)	
Gender		
Male	101	33.3
Female	202	66.7
Education		
Primary school	166	54.8
Junior high school	86	28.3
Senior high school	35	11.6
College and above	16	5.3
Relationship		
Spouse	72	23.8
Children	95	31.3
Children-in-law	18	5.9
Other relatives	13	4.3
Care workers	99	32.7
Social worker	6	2.0
Income (month)		
≤1000 yuan	71	23.4
1001–3000 yuan	117	38.6
3001–5000 yuan	74	24.4
>5000 yuan	41	13.5

According to the exchange rate on April 10, 2018, 1000 yuan = approximately 158.7 USD

In the group of main caregivers with a mean age of 59.0 (SD = 12.7) 66.6% were female. Most caregivers only had attained primary education. Approximately 32.7% of caregivers were care workers who received a salary from a disabled elderly person, followed by the children (31.3%), and spouses (23.8%) of disabled elderly people. Monthly, 38.6% of caregivers earn 1000–3000 yuan, followed by 3001–5000 yuan (24.4%) and less than 1000 yuan (23.4%).

### Disability status of the elderly and caring burden and difficulties of caregivers

As Table 3 shows, the mean BI score was 40.4 (SD = 32.1); thus, respondents in this study were moderately dependent in BADL overall. There were 3 participants with no dependency in BADL; however, they were still classified as disabled people because they were blind people who could serve themselves in BADL but had difficulty dealing with IADLs, which are more complicated. Respondents with complete dependency occupied the highest percentage (33.3%), followed by mild dependency (31.0%) in BADL. The mean FAQ score, which can indicate disability in IADL, was 21.1 (SD = 8.1). The mean FCTI score, which can indirectly reveal the ability to care for the disabled person, was 11.2 (SD = 7.6). The mean ZBI score showed that overall, the main caregivers had a moderate burden in caring for a disabled elderly person.

**Table 3:** Disability status, caring burden and difficulties (N = 303)

Variables	N	%
BI		
Mean(SD)	40.4(32.1)	
No dependency (100 points)	3	1.0
Mild dependency (61–99 points)	94	31.0
Moderate dependency (41–60 points)	49	16.2
Severe dependency (20–40 points)	56	18.5
Complete dependency (0–19 points)	101	33.3
FAQ		
Mean(SD)	21.1(8.1)	
ZBI		
Mean(SD)	27.7(15.0)	
FCTI		
Mean(SD)	11.2(7.6)	

*BI* Barthel Index, *FAQ* Functional Activities Questionnaire, *ZBI* Zarit Burden Interview, *FCTI* Family Caregiver Task Inventory

### Prevalence of needed help and unmet need in ADL tasks

A total of 282 respondents reported at least one unmet need, 93.1% of all the samples. As Table 4 shows, the prevalence ranged from 58.4% (grooming) to 98.3% (shopping and using vehicles) depending on the type of help needed. More than 90% of respondents needed assistance in 6 activities of daily life. These items were shopping, using vehicles, using stairs, assembling affairs, cooking and ambulating. The prevalence of unmet need ranged from 4.6% (financial management) to 77.2% (using vehicles). Five items had a greater than 50% prevalence of unmet need: using vehicles, using stairs, working on a hobby, social interaction and ambulating.

**Table 4:** Prevalence of needed help and unmet need in ADL tasks (*N* = 303)

Items	Needed help (n_1_)	Rate of needed help (%)	Unmet need (n_2_)	Rate of unmet need (%)
Feeding	218	71.9	46	21.1
Dressing	212	70.0	24	11.3
Bathing	234	77.2	83	35.5
Grooming	177	58.4	34	19.2
Toileting	222	73.3	90	40.5
Chair/bed transfer	271	89.4	125	46.1
Ambulating	283	93.4	170	60.1
Using stairs	294	97.0	215	73.1
Financial management	238	78.5	11	4.6
Assembling affairs	293	96.7	26	8.9
Shopping	298	98.3	54	18.1
Working on a hobby	219	72.3	158	72.1
Doing housework	245	80.9	42	17.1
Cooking	289	95.4	33	11.4
Social interaction	235	77.6	147	62.6
Memorizing	207	68.3	24	11.6
Using vehicles	298	98.3	230	77.2

Prevalence of needed help = n_1_/303;Prevalence of unmet need = n_2_/n_1_

### Risk of unmet needs by demographic characteristic of disabled elderly people and caregivers

The mean quantity of unmet needs was 4.98 (SD = 3.17), which ranged from 0 to 16 with a median of 5. Therefore, all respondents can be divided into two groups. One group’s number of unmet needs was no more than 5, and the other’s number of unmet needs was no less than 6. These 2 groups can also be understood in another way: a group with less unmet needs and a group with more unmet needs. A comparison was carried out that used the 2 groups as dependent variables and characteristics, disability status, caring burden and difficulties as independent variables. As shown in Table 5, Wilcoxon test results revealed a significant association between the scores of BI, FAQ, ZBI, FCTI and unmet needs (Z = − 5.330, − 6.342, − 3.073, − 3.131, *p* ≤ 0.01). It was more likely for the elderly people who had worse status in BADL or IADL and whose caregivers had heavier burdens or more difficulty in caring for them to report more unmet needs. The results from the Chi-squared test indicated that medical expenses of disabled elderly people, education, relationships with care-recipients, and income of caregivers were significantly associated with unmet needs (χ^2^ = 17.979, 8.486, 19.705, 13.838, *p* ≤ 0.05, p ≤ 0.01), whereas individuals who spent more on medical expenses and those whose caregivers had lower education, a more distanced relationship or lower income were more likely to report more unmet needs.

**Table 5:** Risk of unmet needs by demographic characteristic [*N* = 303, M (Q_U_, Q_L_), n (%)]

Variables		Quantity of unmet needs≤5	Quantity of unmet needs≥6	Z/χ^2^
Disabled elderly people				
BI^a^		57.5(15.0, 80.0)	25.0(0.0, 55.0)	−5.330^**^
FAQ^a^		17.5(11.0, 26.0)	26.0(20.0, 30.0)	−6.342^**^
Age (year)^a^		83.0(76.0, 88.0)	85.0(79.0, 90.0)	−1.835
Gender	Male	77(47.5)	61(43.3)	0.554
	Female	85(52.5)	80(56.7)	
Marital status	Unmarried	74(45.7)	76(53.9)	2.039
	Married	88(54.3)	65(46.1)	
Living area	Urban	77(47.5)	73(51.8)	5.845
	Suburban	73(45.1)	48(34.0)	
	Rural	12(7.4)	20(14.2)	
Education	Illiterate	61(37.7)	54(38.3)	1.191
	Primary school	39(24.1)	30(21.3)	
	Junior high school	21(13.0)	17(12.1)	
	Senior high school	22(13.6)	18(12.8)	
	College and above	19(11.7)	22(15.6)	
Income (month)	≤1000 yuan	63(38.9)	58(41.1)	0.762
	1001–3000 yuan	29(17.9)	22(15.6)	
	3001–5000 yuan	35(21.6)	34(24.1)	
	>5000 yuan	35(21.6)	27(19.1)	
Medical expense (month)	≤1000 yuan	103(63.6)	74(52.5)	17.979^**^
	1001–3000 yuan	28(17.3)	11(7.8)	
	3001–5000 yuan	12(7.4)	22(15.6)	
	>5000 yuan	19(11.7)	34(24.1)	
Cause of disability	Diseases	115(71.0)	103(73.0)	2.406
	Accidents	25(15.4)	14(9.9)	
	Caducity	22(13.6)	24(17.0)	
Length of disability	≤12 month	18(11.1)	16(11.3)	0.159
	13–24 month	34(21.0)	27(19.1)	
	>24 month	110(67.9)	98(69.5)	
*Main caregivers*				
ZBI^a^		23.5(13.8, 36.0)	30.0(18.0, 41.5)	−3.073^**^
FCTI^a^		8.0(4.0, 14.0)	10.0(7.0, 16.5)	−3.131^**^
Age (year)^a^		60.0(50.0, 70.0)	55.0(55.0, 66.0)	−1.412
Gender	Male	50(30.9)	51(36.2)	0.955
	Female	112(69.1)	90(63.8)	
Education	Primary school	83(51.2)	83(58.9)	8.486^*^
	Junior high school	45(27.8)	41(29.1)	
	Senior high school	20(12.3)	15(10.6)	
	College and above	14(8.6)	2(1.4)	
Relationship	Spouse	52(32.1)	20(14.2)	19.705^**b^
	Children	54(33.3)	41(29.1)	
	Children in law	8(4.9)	10(7.1)	
	Other relatives	4(2.5)	9(6.4)	
	Care workers	41(25.3)	58(41.1)	
	Social worker	3(1.9)	3(2.1)	
Income (month)	≤1000 yuan	38(23.5)	33(23.4)	13.838^**^
	1001–3000 yuan	49(30.2)	68(48.2)	
	3001–5000 yuan	51(31.5)	23(16.3)	
	>5000 yuan	24(14.8)	17(12.1)	

*BI* Barthel Index, *FAQ* Functional Activities Questionnaire, *ZBI* Zarit Burden Interview, *FCTI* Family Caregiver Task Inventory

^*^p ≤ 0.05; ^**^p ≤ 0.01

^a^Do not fit the normal distribution

^b^Use adjusted Chi-squared analysis

### Predictors of unmet needs—Results of binary logistic regression

A binary logistic regression that used the 2 groups as the dependent variables and significant risk factors in the univariate analysis as independent variables was used to identify factors associated with unmet needs among disabled elderly people. The regression model was adjusted for all other variables listed in Table 5**.** Table 6 shows that the likelihood of unmet needs increased with worsening disability status in IADL (OR = 1.079, *p* ≤ 0.01), more distanced relationships with main caregivers (OR = 1.429, *p* ≤ 0.05) and lower incomes of main caregivers (OR = 0.679, p ≤ 0.05).

**Table 6:** Predictors of unmet needs—results of binary logistic regression (N = 303)

Variables	B	S.E.	Adjusted OR	95% C.I. for OR	
				Lower	Upper
Disabled elderly people					
BI	−0.003	0.007	0.997	0.984	1.010
FAQ	0.076	0.026	1.079^**^	1.025	1.136
Medical expense	0.350	0.194	1.419	0.970	2.078
Main caregivers					
ZBI	0.005	0.012	1.005	0.981	1.030
FCTI	0.034	0.024	1.035	0.987	1.085
Education	0.182	0.197	1.200	0.815	1.765
Relationship	0.357	0.144	1.429^*^	1.078	1.895
Income	− 0.387	0.182	0.679^*^	0.475	0.970

All the other variables listed in Table 5 were adjusted, but only significant risk factors in the univariate analysis are listed in this table

*BI* Barthel Index, *FAQ* Functional Activities Questionnaire, *ZBI* Zarit Burden Interview, *FCTI* Family Caregiver Task Inventory

^*^p ≤ 0.05; ^**^ *p* ≤ 0.01

## Discussion

In this study, we found that most disabled elderly people had remained in a situation of low education and low income, which was consistent with several previous studies in China [13, 39, 40]. It was also found that a large percentage of disabled elderly people had suffered from disability for more than 2 years. Hence, it is necessary for China to establish its own long-term care system.

Concordant with a research study in Taiwan [41], middle-aged women were mainly responsible for taking care of the disabled elderly people. This group of caregivers was in a stage of physical deterioration [42], so activities such as moving the elderly person had the potential for injury to the caregiver. Although relatives, especially spouses and children, are still the main caregivers in this study, with the development of the economy, more and more Chinese families have the ability to hire a 24-h care worker to live in their home. However, people with high education refuse jobs as care workers, resulting in a limited number of educated caregivers to improve the quality of life of the disabled elderly person.

The 31.0% prevalence of elderly people with mild dependency in BADL in our study was much lower compared with a national research study in 2010 [6]. In that national study, the prevalence of disabled elderly people with mild dependency was 84.3%, but the instrument used was the Katz Index, which is much simpler than the Barthel Index. The BI has more levels, so the mild dependency in BI may not be equal to that in Katz. Nevertheless, it is interesting that the mildest and most severe levels of dependency occupied the highest or second highest percentage, respectively, and moderate dependency had only a low percentage in both studies. This result may indicate that mild dependency can easily progress to severe or complete dependency without timely intervention. Thus, it is important to give early care and attention to mildly dependent elderly people to slow the progression to complete dependency.

The results of this study showed that 93.1% of disabled elderly people had at least one unmet need. This prevalence was much higher than that shown in previous studies carried out in other regions of the world. Desai found that 20.7% of older Americans had one or more unmet needs in BADL [43]. Rice gave the conclusion that 43.6% of disabled elderly people in 6 states of the United States had unmet needs for assistance [44]. Marc reported that 47.8% of older American Indians had unmet assistance needs [45]. Regarding some Asian regions, the percentage of disabled adults with unmet needs in Taiwan was 44.8% [41], and 18.0% of functionally disabled older Malaysians suffered from unmet needs [46]. Three reasons may explain the huge gap between our study and others in terms of the prevalence of unmet needs. The first is that activities with negative consequences were considered unmet needs in our study. The second is that samples recruited in our research were selected from community electronic health systems, which are more likely to include individuals with special requests. The third is that lifestyles and attitudes of the Chinese may make it possible to have more unmet needs.

The prevalence of unmet needs varied widely in different ADL tasks. Five tasks, using vehicles, using stairs, working on a hobby, social interaction and ambulating, had a rate that was higher than 50%. Hence, a high percentage of disabled elderly people living in communities had unmet needs for activities that required going outside the bedroom and involved spiritual aspects. In the United States, only 11% of disabled elderly people have unmet needs in activities relating to “getting outside” [43], because a single house and cars are common and affordable for most Americans. However, most Chinese elderly people live in apartments located in buildings with several floors, some of which do not have an elevator. Caregivers who are not educated in the care of the disabled may find it hard to assist with activities such as the use of stairs or vehicles without necessary assistive equipment. However, compared with Western countries, equipment that can lift and move patients is still not very popular in China. Many disabled elderly people have no choice but to stay in their rooms or even lie in bed for long periods of time. The lack of outdoor activities may lead to negative effects in these individuals. Feng found that in rural areas of China, 29.3% of the disabled elderly people experienced psychological distress [13]. Quail also reported that unmet IADL needs were significantly associated with elevated psychological distress [20]. However, many Chinese caregivers only considered caring for disabled elderly people as satisfying their physical needs. Spiritual activities such as working on a hobby and social interaction were easily ignored. Therefore, it is urgent for caregivers to change their attitudes and for community institutions to give more professional training to caregivers. Disabled elderly people usually have a feeling of inferiority. They are not confident in communicating with others. Thus, encouragement from their caregivers is necessary. In addition, the construction of facilities for recreation and social interaction in Chinese communities is lagging behind other nations, and the provided facilities are usually not suitable for disabled persons. Therefore, community institutions in China still have considerable room for improvement.

Binary logistic regression findings revealed that disability statuses of IADL, relationships with caregivers and caregiver income were significantly associated with having more unmet needs. Newcomer and Desal shared the same conclusion; unmet needs increased with increases in the number of BADL limitations among disabled elderly Americans [43, 47]. BADL score was also found to be one of the factors that affected unmet needs in Taiwan [41]. Our results did not find an association between unmet needs and BADL scores after adjusting the binary logistic regression but showed that the disabled elderly people who performed worse in IADL were more likely to have more unmet needs. The different results may also be due to the attitudes of Chinese caregivers, which were mentioned above. Caregivers may ignore the importance of IADL needs. Relationships between disabled elderly people and their caregivers were sorted as 1–6 from the closest (spouse) to the most distanced (social workers) in our study. The closer the relationship, the lower the chance of increased unmet needs. It is easy to assume that caregivers with closer relationships to care-recipients would be more attentive and patient in providing better care. Tennstedt did not find an association between income and unmet needs. In contrast, Desai and Drennan reported significant associations [43, 48, 49]. However, only the caregivers’ income was associated with more unmet needs in our study. This association is partly because the satisfaction of caregivers is dependent on money. Additionally, as one of the important parts of caregiving, care workers may do better if they can get a higher salary from care recipients.

Our study has several limitations. The first concerns the cross-sectional design of the study, which could limit its ability to capture the causal relationship among variables. The second comes from the fact that all data were collected through self-reported methods, which may cause information bias. The third is that our samples were recruited only from one region of China, which may cause selection bias. Our future studies will try to collect more samples from different regions, find a better way to improve the precision and multiplicity of analyses and find interventions to decrease the unmet needs of the disabled elderly people.

## Conclusions

In this study, unmet needs for ADL tasks among disabled elderly people are observed to be serious. Disabled elderly people living in communities had a high percentage of unmet needs for activities that require going outside the bedroom and that involve spiritual aspects. Unmet needs increased with worsened disability status in IADL, more distanced relationships with caregivers and lower incomes of caregivers. Government and caregivers should take more useful actions to prevent or reduce unmet needs.

## Additional files

Additional file 1: Demographic Questionnaire. (DOCX 18 kb)

Additional file 2: Barthel Index. (DOC 36 kb)

Additional file 3: Functional Activities Questionnaire. (DOCX 17 kb)

Additional file 4: Zarit Burden Interview. (DOCX 17 kb)

Additional file 5: Family Caregiver Task Inventory. (DOCX 18 kb)

Additional file 6: Unmet Needs Assessment. (DOCX 22 kb)

## Abbreviations

95%CI: 95% confidence intervals
ADL: Activities of daily living
BADL: Basic activities of daily ling
BI: Barthel index
FAQ: Functional activities questionnaire
FCTI: Family caregiver task inventory
IADL: Instrumental activities of daily ling
OR: Odds radios
SD: Standard deviation
SE: Standard error
ZBI: Zarit burden interview
