# Supplementary material for: Unmet needs of activities of daily living among a community-based sample of disabled elderly people in Eastern China: a cross-sectional study
Source: BMC Geriatr. 2018 Jul 11;18:160. doi: 10.1186/s12877-018-0856-6 (PMC6042452; doi:10.1186/s12877-018-0856-6)
Supplement: Supplementary file 1 — Demographic Questionnaire. (DOCX 18 kb) [file 12877_2018_856_MOESM1_ESM.docx]

**Demographic Questionnaire**

| Items | Choices | Answers |
| --- | --- | --- |
| **Disabled Elderly People** | | |
| Gender | 1. □Male 2. □Female |  |
| Date of Birth | (Year) (Month) |  |
| Marital Status | 1. □Unmarried 2. □Married |  |
| Living Area | 1. □Urban 2. □Suburban 3. □Rural |  |
| Education | 1. □Illiterate 2. □Primary School  3. □Junior High School 4. □Senior High School  5. □College and above |  |
| Income(monthly) | 1. □≤1000 yuan 2. □1001~3000 yuan  3. □3001~5000 yuan 4. □＞5000 yuan |  |
| Medical Expense(monthly) | 1. □≤1000 yuan 2. □1001~3000 yuan  3. □3001~5000 yuan 4. □＞5000 yuan |  |
| Cause of Disability | 1. □Diseases 2. □Accidents 3. □Caducity |  |
| Length of Disability | 1. □≤12 months 2. □13~24 months  3. □＞24 months |  |
| **Main Caregivers** | | |
| Gender | 1. □Male 2. □Female |  |
| Date of Birth | (Year) (Month) |  |
| Education | 1. □Primary School 2. □Junior High School  3. □Senior High School 4. □College and above |  |
| Relationship | 1. □Spouse 2. □Children  3. □Children-in-law 4. □Other Relatives  5. □Care Worker 6. □Social Worker |  |
| Income(monthly) | 1. □≤1000 yuan 2. □1001~3000 yuan  3. □3001~5000 yuan 4. □＞5000 yuan |  |
