# Supplementary material for: Unmet needs of activities of daily living among a community-based sample of disabled elderly people in Eastern China: a cross-sectional study
Source: BMC Geriatr. 2018 Jul 11;18:160. doi: 10.1186/s12877-018-0856-6 (PMC6042452; doi:10.1186/s12877-018-0856-6)
Supplement: Supplementary file 2 — Barthel Index. (DOC 36 kb) [file 12877_2018_856_MOESM2_ESM.doc]

**Barthel Index**

| Activities | Rules | Scores |
| --- | --- | --- |
| Feeding | 0=unable  5=needs help cutting, spreading butter, etc., or requires modified diet  10=independent |  |
| Dressing | 0=dependent  5=needs help but can do about half unaided  10=independent (including buttons, zips, laces, etc.) |  |
| Bathing | 0=dependent  5=independent (or in shower) |  |
| Grooming | 0=need to help with personal care  5=independent face/hair/teeth/shaving (implements provided) |  |
| Toileting | 0=dependent  5=needs some help, but can do something alone  10=independent (on and off, dressing, wiping) |  |
| Bowel Control | 0=incontinent (or need to be given enemas)  5=occasional accident  10=continent |  |
| Bladder Control | 0= incontinent (or catheterized and unable to manage alone)  5= occasional accident  10= continent |  |
| Chair/bed Transfer | 0=unable, no sitting balance  5=major help (on or two people, physical), can sit  10=minor help (verbal or physical)  15=independent |  |
| Ambulating | 0=immobile or ＜50 yards  5=wheelchair independent, including corners, ＞50 yards  10=walks with help of one person (verbal or physical) ＞50 yards  15=independent (but may use and aid; for example, stick) ＞50 yards |  |
| Using stairs | 0=unable  5=needs help (verbal, physical, carrying aid)  10=independent |  |
| Total Scores | |  |
