# Supplementary material for: Unmet needs of activities of daily living among a community-based sample of disabled elderly people in Eastern China: a cross-sectional study
Source: BMC Geriatr. 2018 Jul 11;18:160. doi: 10.1186/s12877-018-0856-6 (PMC6042452; doi:10.1186/s12877-018-0856-6)
Supplement: Supplementary file 3 — Functional Activities Questionnaire. (DOCX 17 kb) [file 12877_2018_856_MOESM3_ESM.docx]

**Functional Activities Questionnaire**

**Dependent=3, Requires assistance=2, Has difficulty but does by himself/herself=1, Normal=0, Never did [the activity] but could do now=0, Never did and would have difficulty now=1**

| Items | 0 | 1 | 2 | 3 |
| --- | --- | --- | --- | --- |
| 1. Writing checks, paying bills, balancing checkbook |  |  |  |  |
| 2. Assembling tax records, business affairs, or papers |  |  |  |  |
| 3. Shopping alone for clothes, household necessities, or groceries |  |  |  |  |
| 4. Playing a game of skill, working on a hobby |  |  |  |  |
| 5. Heating water, making a cup of coffee, turning off stove after use |  |  |  |  |
| 6. Preparing a balanced meal |  |  |  |  |
| 7. Keeping track of current events |  |  |  |  |
| 8. Paying attention to, understanding, discussing TV, books, magazine |  |  |  |  |
| 9. Remembering appointment, family occasion, holidays, medications |  |  |  |  |
| 10. Traveling out of neighborhood, driving, arranging to take buses |  |  |  |  |
| Total Scores |  | | | |
