# Supplementary material for: Unmet needs of activities of daily living among a community-based sample of disabled elderly people in Eastern China: a cross-sectional study
Source: BMC Geriatr. 2018 Jul 11;18:160. doi: 10.1186/s12877-018-0856-6 (PMC6042452; doi:10.1186/s12877-018-0856-6)
Supplement: Supplementary file 4 — Zarit Burden Interview. (DOCX 17 kb) [file 12877_2018_856_MOESM4_ESM.docx]

**Zarit Burden Interview**

**(0=NEVER, 1=RERALY, 2=SOMETIMES, 3=QUITE FREQUENTLY, 4=NEARLY ALWAYS)**

| Questions | 0 | 1 | 2 | 3 | 4 |
| --- | --- | --- | --- | --- | --- |
| 1.Do you feel that care recipient asks for more help than he/she needs? |  |  |  |  |  |
| 2.Do you feel that because of the time you spend with care recipient that you don’t have enough time for yourself? |  |  |  |  |  |
| 3.Do you feel stressed between caring for care recipient and trying to meet other responsibilities for your family or work? |  |  |  |  |  |
| 4.Do you feel embarrassed over care recipient’s behavior? |  |  |  |  |  |
| 5.Do you feel angry when you are around care recipient? |  |  |  |  |  |
| 6.Do you feel that care recipient currently affects our relationships with other family members or friends in a negative way? |  |  |  |  |  |
| 7.Are you afraid what the future holds for care recipient? |  |  |  |  |  |
| 8.Do you feel care recipient is dependent on you? |  |  |  |  |  |
| 9.Do you feel strained when you are around care recipient? |  |  |  |  |  |
| 10.Do you feel your health has suffered because you are caring for care recipient? |  |  |  |  |  |
| 11.Do you feel that you don’t have much privacy as you would like because of care recipient? |  |  |  |  |  |
| 12.Do you feel that your social life has suffered because you are caring for care recipient? |  |  |  |  |  |
| 13.Do you feel uncomfortable about having friends over because of care recipient? |  |  |  |  |  |
| 14.Do you feel that care recipient seems to expect you to take care of him/her as if you were the only one he/she could depend on? |  |  |  |  |  |
| 15.Do you think you don’t have enough money to take care of care recipient in addition to the rest of your expenses? |  |  |  |  |  |
| 16.Do you feel that you will be unable to take care of care recipient much longer? |  |  |  |  |  |
| 17.Do you feel that you have lost control of your life because of care recipient? |  |  |  |  |  |
| 18.Do you wish you could leave the care of care recipient to someone else? |  |  |  |  |  |
| 19.Do you feel uncertain about what to do about care recipient? |  |  |  |  |  |
| 20.Do you feel you should be doing more for care recipient? |  |  |  |  |  |
| 21.Do you feel you could do a better job in caring for care recipient? |  |  |  |  |  |
| 22.Overall, how burdened do you feel in caring for care recipient? |  |  |  |  |  |
