# Supplementary material for: Unmet needs of activities of daily living among a community-based sample of disabled elderly people in Eastern China: a cross-sectional study
Source: BMC Geriatr. 2018 Jul 11;18:160. doi: 10.1186/s12877-018-0856-6 (PMC6042452; doi:10.1186/s12877-018-0856-6)
Supplement: Supplementary file 5 — Family Caregiver Task Inventory. (DOCX 18 kb) [file 12877_2018_856_MOESM5_ESM.docx]

**Family Caregiver Task Inventory**

(This is only a translated version, not an official English version. Do not use it directly)

(Not difficult=0, Difficult=1, Very difficult=2)

| Items | 0 | 1 | 2 |
| --- | --- | --- | --- |
| 1. Observe the progression of diseases |  |  |  |
| 2. Help care recipients lead a normal life when they are disabled |  |  |  |
| 3．Help care recipients in their activities of daily livings |  |  |  |
| 4. Learn more knowledge and skills about care recipients’ diseases |  |  |  |
| 5. Deal with potential losses in the future |  |  |  |
| 6. Provide timely assistance to care recipients |  |  |  |
| 7. Supervise care recipients to comply with doctor’s instructions |  |  |  |
| 8. Assess the residual capacity of care recipients |  |  |  |
| 9. Deal with the distressing behavior of care recipients |  |  |  |
| 10. Give more consideration to care recipients’ opinions and preferences |  |  |  |
| 11. Eliminate the feeling of guiltiness when you feel discomfort with care recipients |  |  |  |
| 12. Vent emotions appropriately |  |  |  |
| 13. Distinguish feelings about diseases and feelings about care recipients |  |  |  |
| 14. Eliminate the feeling of uncertainty about your caring skills |  |  |  |
| 15. Relieve nervous feelings towards care recipients |  |  |  |
| 16. Estimate assistance and services in the future |  |  |  |
| 17. Your family members’ help is your first choice when you are in trouble |  |  |  |
| 18. Deal with bad feelings from family members who cannot help you in time |  |  |  |
| 19. Maintain the family is an integral part of making decisions |  |  |  |
| 20. Contact with medical staff |  |  |  |
| 21. Have a creative life |  |  |  |
| 22. Avoid serious physical exertion |  |  |  |
| 23. Have a future plan |  |  |  |
| 24. Adjust your own daily livings |  |  |  |
| 25. Make up for disturbed sleep |  |  |  |
| Total scores |  | | |
