# Supplementary material for: Unmet needs of activities of daily living among a community-based sample of disabled elderly people in Eastern China: a cross-sectional study
Source: BMC Geriatr. 2018 Jul 11;18:160. doi: 10.1186/s12877-018-0856-6 (PMC6042452; doi:10.1186/s12877-018-0856-6)
Supplement: Supplementary file 6 — Unmet Needs Assessment. (DOCX 22 kb) [file 12877_2018_856_MOESM6_ESM.docx]

**Unmet BADL Needs Assessment**

| Items | Questions (During the past month) | Judgement |
| --- | --- | --- |
| Feeding | 1. Do you receive someone’s help with feeding?  □Yes □No  2. Do you need more help with feeding? (Is the present assistance enough?)  □No □Yes  3. Were there times you were unable to eat when you were hungry because no one was available to help you have food?  □No □Yes |  |
| Dressing | 1. Do you receive someone’s help with dressing?  □Yes □No  2. Do you need more help with dressing? (Is the present assistance enough?)  □No □Yes  3. Did you experience discomfort because you were unable to get assistance to change your dirty or wet cloths as soon as possible?  □No □Yes |  |
| Bathing | 1. Do you receive someone’s help with bathing?  □Yes □No  2. Do you need more help with bathing? (Is the present assistance enough?)  □No □Yes  3. Did you experience discomfort because you were unable to get assistance to bathe as often as possible?  □No □Yes  4. Did you experience a scald caused by bathing with water that was too hot?  □No □Yes |  |
| Grooming | 1. Do you receive someone’s help with grooming?  □Yes □No  2. Do you need more help with grooming? (Is the present assistance enough?)  □No □Yes  3. Did you experience discomfort with your face/teeth/hair because you were unable to get assistance to groom as often as possible?  □No □Yes |  |
| Toileting | 1. Do you receive someone’s help with toileting?  □Yes □No  2. Do you need more help with toileting? (Is the present assistance enough?)  □No □Yes  3. Were there times you had no choice but to hold back urine for a long time because no one was available to help you go to the restroom?  □No □Yes  4. Did you wet or soil yourself because you did not have help getting to the restroom?  □No □Yes |  |
| Chair/bed transferring | 1. Do you receive someone’s help with chair/bed transferring?  □Yes □No  2. Do you need more help with chair/bed transferring? (Is the present assistance enough?)  □No □Yes  3. Were there times you had no choice but to lie on the bed for a long time because no one was available to help you get out of bed?  □No □Yes  4. Have you ever had pressure sores?  □No □Yes |  |
| Ambulating | 1. Do you receive someone’s help with ambulating?  □Yes □No  2. Do you need more help with ambulating? (Is the present assistance enough?)  □No □Yes  3. Were there times you had no choice but to stay in the bedroom for a long time because no one was available to help you walk outside?  □No □Yes |  |
| Using stairs | 1. Do you receive someone’s help with using stairs?  □Yes □No  2. Do you need more help with using stairs? (Is the present assistance enough?)  □No □Yes  3. Were there times you had no choice but to stay on the same floor for a long time because no one was available to help you go downstairs?  □No □Yes |  |

**Unmet IADL Needs Assessment**

| Items | Questions (During the past month) | Judgement |
| --- | --- | --- |
| Financial management | 1. Do you receive someone’s help with financial management?  □Yes □No  2. Do you need more help with financial management? (Is the present assistance enough?)  □No □Yes  3. Were there times you lost money or overspent because no one was available to help you do financial management?  □No □Yes |  |
| Assembling affairs | 1. Do you receive someone’s help with assembling affairs?  □Yes □No  2. Do you need more help with assembling affairs? (Is the present assistance enough?)  □No □Yes  3. Did you experience chaos in everyday life because you were unable to get assistance to assemble affairs?  □No □Yes |  |
| Shopping | 1. Do you receive someone’s help with shopping?  □Yes □No  2. Do you need more help with shopping? (Is the present assistance enough?)  □No □Yes  3. Did you experience inconvenience due to lack of daily supplies because you were unable to get assistance to do shopping?  □No □Yes |  |
| Working on a hobby | 1. Do you receive someone’s help with your hobby?  □Yes □No  2. Do you need more help with your hobby? (Is the present assistance enough?)  □No □Yes  3. Were there times you felt very bored because no one was available to help you do recreational activities?  □No □Yes |  |
| Doing Housework | 1. Do you receive someone’s help with your housework?  □Yes □No  2. Do you need more help with your housework? (Is the present assistance enough?)  □No □Yes  3. Were there times you had no choice but to live in a dirty environment because no one was available to help you do housework?  □No □Yes |  |
| Cooking | 1. Do you receive someone’s help with cooking?  □Yes □No  2. Do you need more help with cooking? (Is the present assistance enough?)  □No □Yes  3. Did you experience discomfort because you often had to eat food that did not meet your taste?  □No □Yes |  |
| Social interaction | 1. Do you receive someone’s help with social interaction?  □Yes □No  2. Do you need more help with social interaction? (Is the present assistance enough?)  □No □Yes  3. Were there times you had no choice but to live alone for a long time because no one was available to help you go outside to chat with others?  □No □Yes |  |
| Memorizing | 1. Do you receive someone’s help with memorizing?  □Yes □No  2. Do you need more help with memorizing? (Is the present assistance enough?)  □No □Yes  3. Were there times you forgot to take medicine or misused medicine because no one was available to remind you to take the medicine?  □No □Yes |  |
| Using vehicles | 1. Do you receive someone’s help with using vehicles?  □Yes □No  2. Do you need more help with using vehicles? (Is the present assistance enough?)  □No □Yes  3. Were there times you had no choice but to stay home when there was an urgent issue that needed to be dealt with outside because no one was available to take you to travel some distance?  □No □Yes |  |
